# Clindamycin Resistance among Staphylococcus Aureus Isolated at Mbarara Regional Referral Hospital, in South Western Uganda

**DOI:** 10.9734/BMRJ/2014/10572

**Published:** 2014-07-23

**Authors:** Bashir Mwambi, Jacob Iramiot, Freddie Bwanga, Marthae Nakaye, Herbert Itabangi, Joel Bazira

**Affiliations:** 1 Department of Microbiology, Mbarara University of Science and Technology, P.O.Box 1410 Mbarara, Uganda.; 2 Department of Medical Microbiology Makerere University College of Health Sciences P. O Box 7072 Kampala, Uganda.; 3 MBN Clinical Laboratories, Uganda.

**Keywords:** Staphylococcus aureus, clindamycin, phenotypic, genetic, resistance, d-test

## Abstract

**Aims:**

The study was conducted to determine the prevalence of Clindamycin (CL) resistance and antimicrobial susceptibility among clinical isolates of *Staphylococcus aureus* (*S. aureus*) from Mbarara Regional Referral Hospital (MRRH) in Southwestern Uganda.

**Study Design:**

Laboratory based cross sectional study.

**Place and Duration of the Study:**

The study was conducted at the Microbiology department of Mbarara Regional referral hospital between November 2012 and December 2013.

**Methodology:**

In our study, we recruited 300 *S. aureus* isolates that were stored in the laboratory and were obtained from different clinical samples. The isolates were tested for antimicrobial susceptibility by phenotypic methods and for the genotypic expression of Macrolide Lincosamide StreptograminB (MLS_B_) resistance genes (*ermA*, *ermB*, *ermC*, and *msrA*). The D-test was also performed.

**Results:**

Phenotypically, a total of 109 (36%) *S. aureus* isolates were resistant to CL, of which 9 (3%) were constitutively resistant while 100 (33.3%) were inducibly resistant. Genotypicaly, 134/300 (44.7%) isolates possessed at least one of the MLS_B_ resistance genes. 23/300 (7.7%) tested positive for *ermB*, 98/300 (32.7%) tested positive for the *ermC* and 43/300 (14.3%) tested positive for the *msrA* genes with none possessing the *ermA* gene. Isolates were highly resistant to Sulfamethoxazole/trimethoprim, Erythromycin and Oxacillin with moderate resistance to Vancomycin and Imipenem and least resistance to Linezolid

**Conclusion:**

*S. aureus* resistance to CL was high in this set up. There was also high resistance to Sulfamethoxazole/trimethoprim, Erythromycin and Oxacillin but low resistance to Linezolid.

## 1. INTRODUCTION

### 1.1 Background

*Staphylococcus aureus* (*S. aureus*) is a common cause of both community and nosocomial acquired infections ranging from minor skin infections to life threatening conditions such as endocarditis, pneumonia and septicaemia. Increasing antimicrobial drug resistance in *S. aureus* is one of the major concerns [1]. Traditionally meticillin resistant *S. aureus* (MRSA) has been considered a nosocomial pathogen and vancomycin considered as drug of choice. However, vancomycin usage is associated with considerable side effects and cost. Moreover overuse of vancomycin has led to the emergence of staphylococcal strains with reduced susceptibility to it [2]. 

Unlike hospital acquired MRSA, the Community acquired MRSA (CA-MRSA) are known to be sensitive to drugs other than vancomycin, such as, ciprofloxacin, sulphamethoxazole-trimethoprim (SXT) and clindamycin (CL). Low cost, fewer severe side effects, availability of oral and parenteral forms, lack of need for renal adjustments, good tissue penetration and ability to directly inhibit toxin production are the advantages of CL. Furthermore, CL is a useful choice in cases of penicillin allergy [3]. Because of an increased use of CL, development of resistance especially inducible resistance has emerged and this has caused a major burden to its usage. 

Bacterial resistance to this group may be expressed through different mechanisms including target site modification, macrolide efflux pump and enzymatic antibiotic inactivation [4]. Modification of the ribosomal target is encoded by the *erm* genes that cause production of methylase enzymes which reduce binding of the drug to the rRNA target, the macrolide efflux pump is by the efflux proteins of the ATP-binding-cassette (ABC) transporter super family that confer acquired macrolide resistance encoded by plasmid borne *msrA* genes, while enzymatic antibiotic inactivation of maclorides in *S. aureus* is by producing phosphotransferases encoded by *mph(C)* genes and inactivation of lincosamides is by Lincosamide nucleotidyl transferases encoded by *lnu(A)*. However, enzymatic inactivation of antimicrobials is common with enterobacteriacae and rare in Gram positive bacteria [4]. 

The resistance can be either constitutive or inducible. If the *erm* genes are consistently expressed, isolates show in vitro resistance to erythromycin (ER), CL, and to other members of MLS_B_, known as constitutive resistance phenotype. In case of inducible resistance, the *erm* genes require an inducing agent to express resistance to CL. ER can act as a strong inducer of methylase synthesis. These isolates known as inducible resistance phenotype show in vitro resistance to ER and susceptibility to CL. CL therapy in this phenotype can lead to clinical failure [5].

*S. aureus* can also develop isolated macrolide resistance based on presence of an efflux pump, encoded by the *msrA* gene which leads to resistance to macrolides and type B streptogramins but not to lincosamides. These isolates known as MS phenotype also show in vitro resistance to ER and susceptibility to CL same as in inducible resistance phenotype, but CL therapy can be safely given in infections with this phenotype and there is no risk of clinical failure. 

Therefore, it is important to differentiate these two mechanisms of resistance. CL is a good option for managing MRSA but the rate of both inducible and constitutive resistance has to be ascertained, as it varies by geographical location and bacterial species. So the aim of this study was to assess the prevalence of inducible and constitutive CL resistance in clinical isolates of *S.aureus*. 

While there are many reports on inducible resistance of *S. aureus* in developed as well as developing countries [6–9], there is limited data about CL resistance in Uganda and in the region only one study reported on inducible CL resistance in Tanzania by Mshana et al*.* [10]. The current study was conducted in order to ascertain the prevalence of *S. aureus* resistance to CL among isolates of *S. aureus* from MRRH Southwestern Uganda and to find alternative antibiotics for management of *S. aureus*.

## 2. MATERIALS AND METHODS

### 2.1 Study Site

The study was conducted at Mbarara Regional Referral Hospital (MRRH) Microbiology Laboratory from November 2012 to December 2013. 300 *Staphylococcus aureus (S. aureus)* isolates from clinical specimens from all the wards at MRRH were included in this laboratory based cross sectional study.

### 2.2 Laboratory Procedures

*S. aureus* isolates retrieved in the laboratory had been obtained from different specimens including blood, cerebral spinal fluid (CSF), ear swab, high vaginal swab (HVS), nasal swab, pus swab, throat swab, urethral swab, urine, and wound swabs. Isolates were subcultured on blood agar and nutrient agar by streaking method. The culture plates were checked for growth after 18 to 24 hours of incubation at 37°C [11]. 

Plate reading, Gram stain and biochemical tests (catalase, coagulase and DNAase) were performed to identify *S. aureus*. The identified colonies were inoculated onto Mannitol salt agar (MSA) medium to obtain pure cultures of *S. aureus* [11]. 

Drug susceptibility testing was performed for the following antimicrobials {SXT (25μg), ER (15 μg/mL), oxacillin (1μg/mL), imipenem (5μg/mL), vancomycin (30μg/mL), linezolid (30μg/mL) and CL (2μg/mL)}. The Kirby-bauer method for performing disk diffusion technique to test antimicrobial susceptibility was used [12] and results interpreted according to Clinical Laboratory Standard Institute (CLSI) guidelines for standard performance of antimicrobial disk susceptibility tests 2008 [13]. Inducible CL resistance was determined using the D- test. The D-test was performed on isolates that were resistant to ER but sensitive to CL by placing both CL and ER disks 15 mm apart from the centre on the Mueller hinton agar. Plates were read after 18 to 24 hours of incubation at 35°C. Flattening on the side of ER was read as inducible CL resistance while a zone of clearance towards the side of ER was read as CL sensitive as described by Fiebelkorn et al. [14]. 

Genotypic resistance to CL was determined by the Polymerase chain reaction (PCR) for *ermA, ermB, ermC,* and *msrA* genes. The genes were amplified with a single-plex PCR (sPCR) using specific primers [9, 15]. DNA was extracted from bacteria by the heat method through suspending colonies of *S. aureus* in 100ul digestion buffer (10mM Tris-HCl, pH 8.0, 0.45% Triton X-100, and 0.45% Tween 20) in 2-ml eppendorf tubes. The tubes containing *S. aureus* suspension were vortexed and then heated at 100°C (in a dry block heater) for 10 min and then cooled on ice and centrifuged for 15 min at 14,000*g* to pellet the cell debris. At this point, negative (PCR water) and positive (*S*. *aureus* RN1551-*ermA*, *S*. *aureus* RN2442-*ermC*, *S*. *aureus* RN4220-*msrA*, and *S*. *aureus* 6520-*ermB*) controls were included as described by Nicola et al. [6]. A 2ul aliquot of each supernatant containing extracted DNA was used as templates for PCR to amplify *ermA*, *ermB*, *ermC* and *msrA* genes (Table 1). For each sPCR the reaction mixture contained; Templates for DNA, forward and reverse primers, deoxynucleoside triphosphates (dNTPs) in equal amounts; PCR buffer, *Taq* polymerase, magnesium Chloride and Molecular grade water.

### 2.3 Quality Control

*S. aureus* ATCC 25923 was used as the control strain for identification and susceptibility tests.

### 2.4 Data Analysis

Data was entered and organized in Microsoft excel and was then imported into STATA ver11 statistical analytical tool where it was analysed and interpreted with Odds ratios, Chi- square test and their *P*-values at 95% CI and finally presented in tables and bar graphs drawn in Microsoft Excel. Phenotypic and genotypic methods were compared using a two by two table with the Kappa statistics, and the *P*-value at .05 was considered significant.

## 3. RESULTS AND DISCUSSION

**Figure 1:**
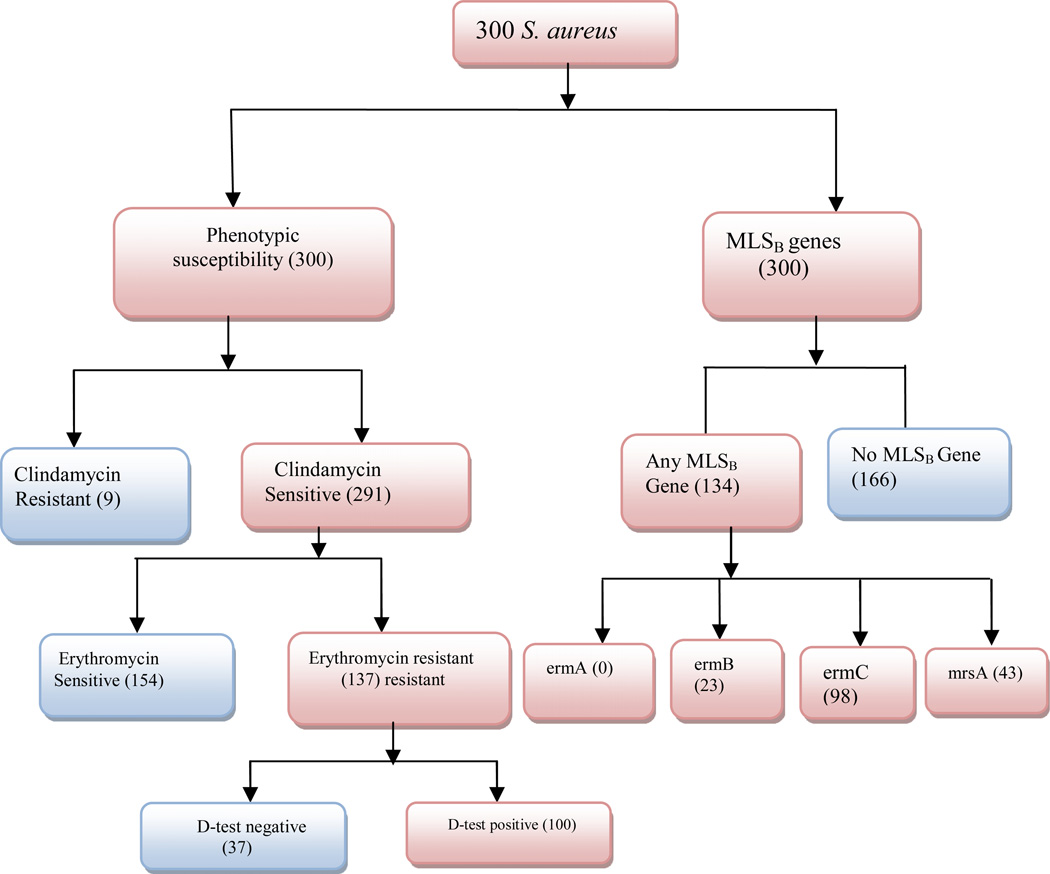
Study profile showing both Phenotypic and genotypic detection of *S. aureus* resistance to CL

The study included 300 *S. aureus* which were collected from 168 female and 132 male patients. The frequency of samples from which *S. aureus* were isolated was; blood (164, 54.67%), CSF (3, 1%), ear swab (6, 2%), HVS (29, 9.67%), nasal swab (11, 3.67%), pus swab (42, 14%), throat swab (1, 0.33%), urethral swab (2, 0.67%), urine (33, 11.00%), and wound swabs (9, 2.99%) and were all tested for resistance to CL by genetic and phenotypic methods (Fig. 1).

### 3.1 Phenotypic Detection of CL Resistance

Phenotypically, a total of 109 (36.3%) *S. aureus* isolates were resistant to clindamycin. Of these, 9 (3%) isolates were constitutively resistant while 100 (33.3%) were inducibly resistant (D-test positive).

### 3.2 Genotypic Detection of Resistance

**Figure 2:**
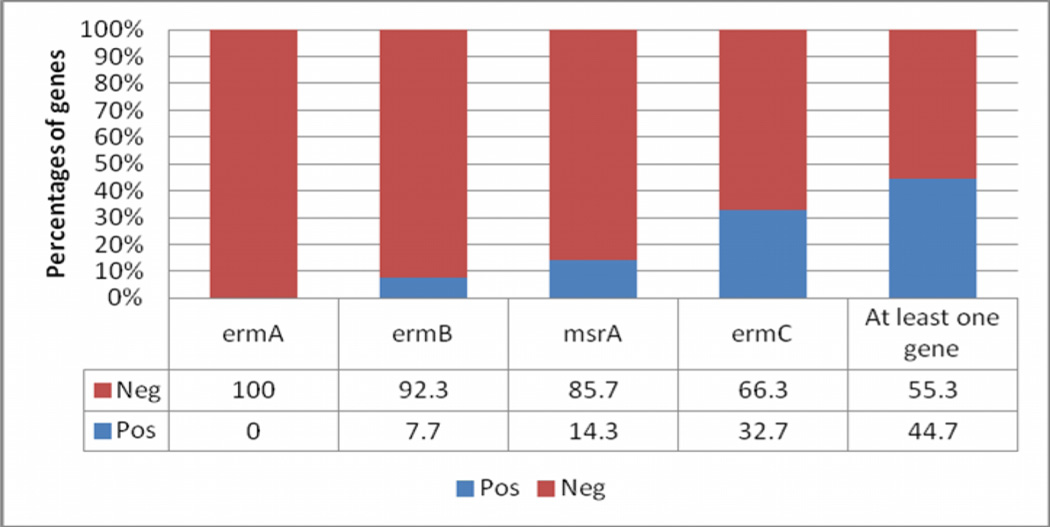
A graph showing the patterns of expression of MLSb resistance genes

Genotypically, 134/300 (44.7%) isolates carried at least one of the MLSB resistance genes with all testing negative for the *ermA* gene (amplicon size 190bp), 23/300 (7.7%) positive for *ermB* (amplicon size 142bp)*,* 98/300 (32.7%) positive for the *ermC* (amplicon size 299bp) and 43/300 (14.3%) positive for the *msrA* genes (amplicon size 163bp). (Fig. 2).

### 3.3 Comparison of Phenotypic and Genotypic Methods

Phenotypic methods detected 109 *S. aureus* strains resistant to CL while genetic method identified more (134) strains carrying at least one of the resistance genes. Of the 134 isolates that carried at least one CL resistance gene, 80 (59.71%) were phenotypically resistant to CL while 54 (40.29%) were sensitive. Of the 166 isolates that did not carry any of the resistance genes, 29 (17.47%) were CL resistant while 137 (82.53%) were sensitive to CL (Table 2). 

The genotypic method for detection of resistance was superior (detected 44.67%, CI=44.51%–44.83%) over detection of resistance with phenotypic methods (detected 36%, CI=35.85%–36.15%), (Z=2.25, *P*-Value=.01), but 29 isolates were phenotypically resistant yet did not express any of the MLS_B_ genes and there were high chances (6.79 times) of identifying an isolate as phenotypically resistant to CL as well as carrying any of the resistance gene (OR 6.79, CI 3.67–11.37 and *P*-value<.001).

### 3.4 Antimicrobial Susceptibility Patterns of *S. aureus*

**Figure 3:**
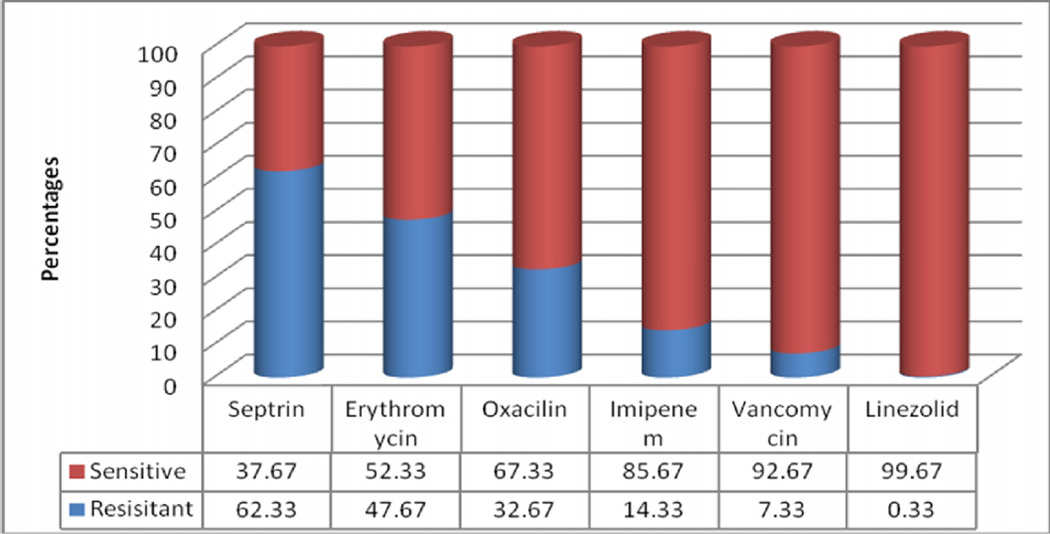
Showing sensitivity patterns of *S. aureus* isolates to different antimicrobials at MRRH

Generally the rate of antimicrobial resistance varied among antimicrobials ranging from SXT 187 (62.33%), ER 143 (47.67%), oxacillin 98 (32.67%), imipenem 43 (14.33%), vancomycin 22 (7.33%) and linezolid 1 (0.33%) (Fig. 3).

## 4. DISCUSSION

Information from this study showed phenotyically an overall prevalence of 36% (108/300) *S. aureus* isolates resistant to clindamycin. Of these, 3% (9/300) isolates were constitutively resistant while 33% (99/300) were inducibly resistant (D-test positive). Similar studies have been conducted including one in India that reported a prevalence rate of 54% *S. aureus* resistance to CL of which 12% constitutively resistant and 43% Inducibly resistant [16]. Still in India Shantala et al. 2011 reported prevalence rate of 43.15% *S. aureus* resistance to CL with 18.26% (42/230) constitutively resistant and 24.89 % (57/230) inducibly resistant [17]. Another study in Turkey reported a prevalence of 45.2% (399/883) CL resistance to *S. aureus,* of which 25.4% (224/883) were the constitutive resistance phenotype and 19.8% (175/883) were the inducible resistance phenotype [18]. The studies in India and Turkey show higher prevalence compared to that obtained in the current study at MRRH and much higher with constitutive resistance. However, the prevalence in this study was higher than that previously obtained in Tanzania (13%) by Mshana et al. [10]. 

In India and Turkey, the use of CL is higher compared to MRRH and this can explains the higher rates of constitutive CL resistance. The use of CL to treat D-test positive strains causing either minor or severe infections may lead to development of constitutive resistant strains which can then be spread in the population creating pressure to CL therapies. But the increase compared to that in 2009 in Tanzania could be explained by an increased use of CL in the region over time. 

Genotypicaly, a total of 134/300 isolates expressed at least one of the MLS_B_ resistance genes giving a prevalence of (44.7%). Amongst these, none tested positive for the *ermA.* This could be due to the fact *ermA* gene being more prevalent in Coagulase Negative Staphylococci (CoNS) [19] yet in this study we only tesed Coagulase postive *S. aureus*. In this study, *ermC* was reported prevalent and this was consitent to that of Thakker-Varia et al. [19] but contradicted that of a report by Nizami et al. [20]. 

Although the genotypic method detected more resistant strains than phenotypic method, the 29 isolates that were phenotypically resistant but did not express any of the MLS_B_ genes cannot allow us draw a final conclusion as Genotypic methods being superior. Nizami et al. 2012 also reported a similar difference between the two methods with a 48.7% resistance to CL by phenotypic methods, while the ratio was 55.4% by multiplex PCR assay and in 34 isolates, no MLS_B_ resistance genes was detected, although they were detected as resistant to CL phenotypically [20]. The discrepancy could have been caused by other genes responsible for the resistance and were not included in the assay.

Sulfamethaxazole/trimethoprim, a more convenient alternative to CL because it requires twice-a-day administration, low cost and "it doesn't taste too bad," showed the worst performance at 62.33% and this could be explained by the overuse and miss use of the antimicrobial in the region. While for other antimicrobials the prescription rates in the region match the resistance rates shown by the antimicrobials; erythromycin at 47.67%, oxacillin at 32.67%, imipenem at 14.33%, vancomycin at 7.33% and linezolid at 0.33% respectively. This indicates resistance rate increases with increasing use of the antimicrobial.

## 5. CONCLUSION

At resistance rates of 36% phenotypically and 44.7% genotypically, leaves CL to be used only after testing for susceptibility when treating MRSA. Empirically, Linezolid should be the drug of choice but also included in a sensitivity panel of antimicrobial to be performed. Sulfamethaxazole/trimethoprim and erythromycin should not be used in management of *S. aureus* infections empirically with the high resistance expressed by *S. aureus* to them. In the study, molecular methods for testing resistance were superior over phenotypic methods with the *ermC* gene being more expressed amongst Inducible resistant strains of CL and could be the gene responsible for inducible resistance. 

## Figures and Tables

**Table 1:** Primer designing for *ermA, ermB, ermC* and *msrA*, PCR conditions and the amplified product size

Gene	Primer designs	Annealing temperature	Annealing time	Size of amplified product (bp)
*ermA*	5’-AAG CGG TAA ACC CCT CTG A-3’ F 5’-TTC GCA AAT CCC TTC TCA AC-3’ R	45°C	30 seconds	190
*ermB*	5’-CTATCTGATTGTTGAAGAAGGATT-3’ F 5’-GTTTACTCTTGGTTTAGGATGAAA-3’ R	45°C	30 seconds	142
*ermC*	5’-AAT CGT CAA TTC CTG CAT GT-3’ F 5’-TAA TCG TGG AAT ACG GGT TTG-3’ R	45°C	30 seconds	299
*msrA*	5’-TCCAATCATTGCACAAAATC-3’ F 5’-AATTCCCTCTATTTGGTGGT-3’ R	45°C	30 seconds	163

Note: F=forward primer and R=reverse primer

**Table 2:** Comparison of expression MLS_B_ resistance genes with phenotypic resistance to CL

Any resistance gene present	Phenotypic susceptibility to CL	
	Resistant	Sensitive
Positive (n:134)	80	54
Negative (n:166)	29	137
Total (n:300)	109	191
